# Contrasting effects of filamin A and B proteins in modulating filovirus entry

**DOI:** 10.1371/journal.ppat.1011595

**Published:** 2023-08-16

**Authors:** Ariel Shepley-McTaggart, Jingjing Liang, Yang Ding, Marija A. Djurkovic, Valeriia Kriachun, Olena Shtanko, Oriol Sunyer, Ronald N. Harty

**Affiliations:** 1 Department of Pathobiology, School of Veterinary Medicine, University of Pennsylvania, Philadelphia, Pennsylvania, United States of America; 2 Host-Pathogen Interactions, Texas Biomedical Research Institute, San Antonio, Texas, United States of America; University of Texas Medical Branch / Galveston National Laboratory, UNITED STATES

## Abstract

Ebola (EBOV) and Marburg viruses (MARV) cause severe hemorrhagic fever associated with high mortality rates in humans. A better understanding of filovirus-host interactions that regulate the EBOV and MARV lifecycles can provide biological and mechanistic insight critical for therapeutic development. EBOV glycoprotein (eGP) and MARV glycoprotein (mGP) mediate entry into host cells primarily by actin-dependent macropinocytosis. Here, we identified actin-binding cytoskeletal crosslinking proteins filamin A (FLNa) and B (FLNb) as important regulators of both EBOV and MARV entry. We found that entry of pseudotype psVSV-RFP-eGP, infectious recombinant rVSV-eGP-mCherry, and live authentic EBOV and MARV was inhibited in filamin A knockdown (FLNaKD) cells, but was surprisingly enhanced in filamin B knockdown (FLNbKD) cells. Mechanistically, our findings suggest that differential regulation of macropinocytosis by FLNa and FLNb likely contributes to their specific effects on EBOV and MARV entry. This study is the first to identify the filamin family of proteins as regulators of EBOV and MARV entry. These findings may provide insight into the development of new countermeasures to prevent EBOV and MARV infections.

## Author summary

Filoviruses utilize host proteins to successfully infect cells and complete their lifecycles. Identification of host proteins that regulate the EBOV and MARV lifecycles may allow us to find possible therapeutic targets to protect against these deadly viruses. Here, we identified actin-binding cytoskeletal proteins filamin A and B as regulators of EBOV and MARV entry. We show that filamin A is a positive regulator while filamin B is a negative regulator of both EBOV and MARV entry. In sum, our findings reveal a role for host cytoskeletal-associated filamin proteins in the entry stage of the filovirus lifecycle, potentially identifying filamin proteins as broad spectrum therapeutic targets for prevention and treatment of EBOV and MARV.

## Introduction

EBOV and MARV remain global public health threats that warrant urgent development of antiviral therapeutics [1–5]. The filoviruses target many cell types, including monocytes, macrophages, and dendritic cells, and viral entry and uptake is mediated by the surface glycoprotein via the process of macropinocytosis [4,6–10]. As host proteins also play critical roles in regulating (positively or negatively) filoviral entry, a better understanding of the interplay between the virus and host may enable us to identify therapeutic targets to combat these deadly pathogens.

While much has been learned regarding the molecular aspects of filovirus entry through the use of surrogate viruses and assays, a more comprehensive understanding of the role of host proteins in both positive and negative regulation of early stages of the filovirus lifecycle is needed. For example, positive regulators such as T-cell Ig mucin domain 1 (TIM-1) signal to trigger the macropinocytosis program required to internalize EBOV virions after eGP attachment, while host receptor NPC1 is required to trigger fusion of the viral and endolysosomal membranes. Additionally, the Tyro3 receptor tyrosine kinase family-Axl, Dtk, and Mer-in have been shown to be involved in entry of both EBOV and MARV [11–14].

The plasma membrane is the site of entry for both EBOV and MARV, and thus the dynamics of the plasma membrane and its associated cytoskeletal proteins will likely play key roles in regulating this early stage of filovirus infection. The filamin proteins comprise a family of three members, filamin A (FLNa), B (FLNb) and C (FLNc), that localize in part to the plasma membrane. The N-terminal region of filamins contains an actin-binding domain, followed by a rod-like domain consisting of 24 tandem repeats that function in crosslinking cortical actin filaments into a dynamic, three-dimensional structure. These proteins also function as molecular scaffolds by connecting numerous functionally diverse proteins [15–17]. The filamin proteins are known to connect the actin cytoskeleton with several cellular receptors, such as dopamine D2 and androgen receptors on the plasma membrane, as well as numerous ß integrin proteins to the cytoskeleton for cell mechanoprotection [18]. FLNa and FLNb also differentially regulate the RhoA GTPase, which is directly involved in promoting macropinocytosis, the process in which EBOV enters cells [7,19–21]. Additionally, while FLNa is ubiquitously expressed in many cell types, FLNb is highly expressed in vascular endothelial cells, a cell type often targeted during filovirus infection [15,22–26]. Although the actin cytoskeleton plays a key role in filoviral entry [7,20,27,28], the role of the actin-binding filamin proteins in the EBOV and MARV lifecycles remains to be determined.

Here, we report on a previously undescribed role for FLNa and FLNb in the filovirus lifecycle: eGP and mGP-meditated entry of EBOV and MARV, respectively. We used pseudotype VSV-RFP-eGP, replication-competent recombinant rVSV-mCherry-eGP, and authentic EBOV and MARV to transduce/infect HT-1080 WT, filamin A knockdown (FLNaKD), and filamin B knockdown (FLNbKD) cells to assess the role of filamin A and B proteins in entry/infectivity. Our findings indicated that knockdown of FLNa inhibited viral infectivity, suggesting FLNa is an important positive regulator of viral entry. In contrast, knockdown of FLNb enhanced viral infectivity, suggesting that expression of FLNb may restrict viral entry. We corroborated these findings using an siRNA approach to knockdown endogenous FLNa or FLNb in HEK293T cells followed by quantification of pseudotype VSV-RFP-eGP entry. Importantly, we showed that siRNA knockdown of endogenous FLNa in primary human macrophages resulted in a significant decrease in both EBOV and MARV infectivity. Toward the mechanism of action, we used flow cytometry and confocal microscopy to show that the filamin proteins regulate macropinocytosis to potentially impact macropinocytosis-mediated entry of EBOV and MARV. Our findings imply that FLNa and FLNb proteins affect filovirus entry inversely by modulating macropinocyctosis. In sum, we identified filamin proteins as novel regulators of both EBOV and MARV entry, and thus filamins may serve as broad-spectrum therapeutic targets to prevent infectivity and reduce transmission.

## Results

### Filamin proteins regulate infectivity of authentic EBOV and MARV

**Fig 1:**
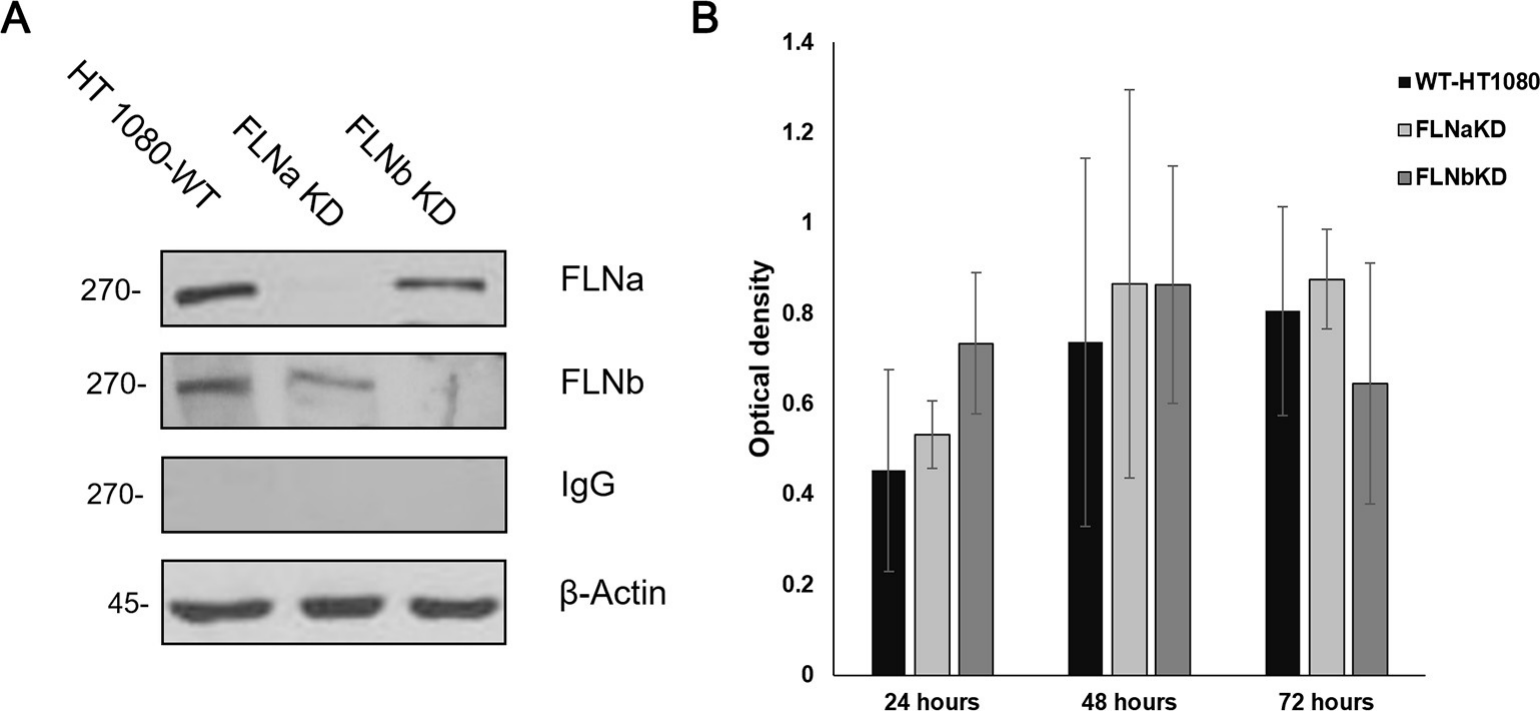
**Analysis of FLN KD cells** (A) Representative Western blot showing stable shRNA knockdown of FLNa in FLNaKD cells (lane 2) and FLNb in FLNbKD cells (lane 3). Both FLNa and FLNb are expressed in WT HT-1080 cells (lane 1). (B) MTT assay showing no significant growth rate or proliferation differences between WT and the filamin KD cells at 24, 48, and 72 hours post-seeding. Statistical analysis of 4 independent experiments using 2-sample student t-test is shown.

To investigate whether filamin A and/or B proteins regulate filoviral infectivity, we utilized HT-1080 cell lines in which endogenous FLNa or FLNb was knocked down using an shRNA approach [15]. We confirmed stable shRNA knockdown of FLNa and FLNb in FLNaKD and FLNbKD cells, respectively, with immunoblotting using anti-FLNa and anti-FLNb antibodies (Fig 1A). We used an MTT assay to evaluate any potential differences in growth rate across the cell types. We found that the FLNa and FLNb knockdown cells had no significant growth rate or proliferation difference from those of the WT parental cell line (Fig 1B).

**Fig 2:**
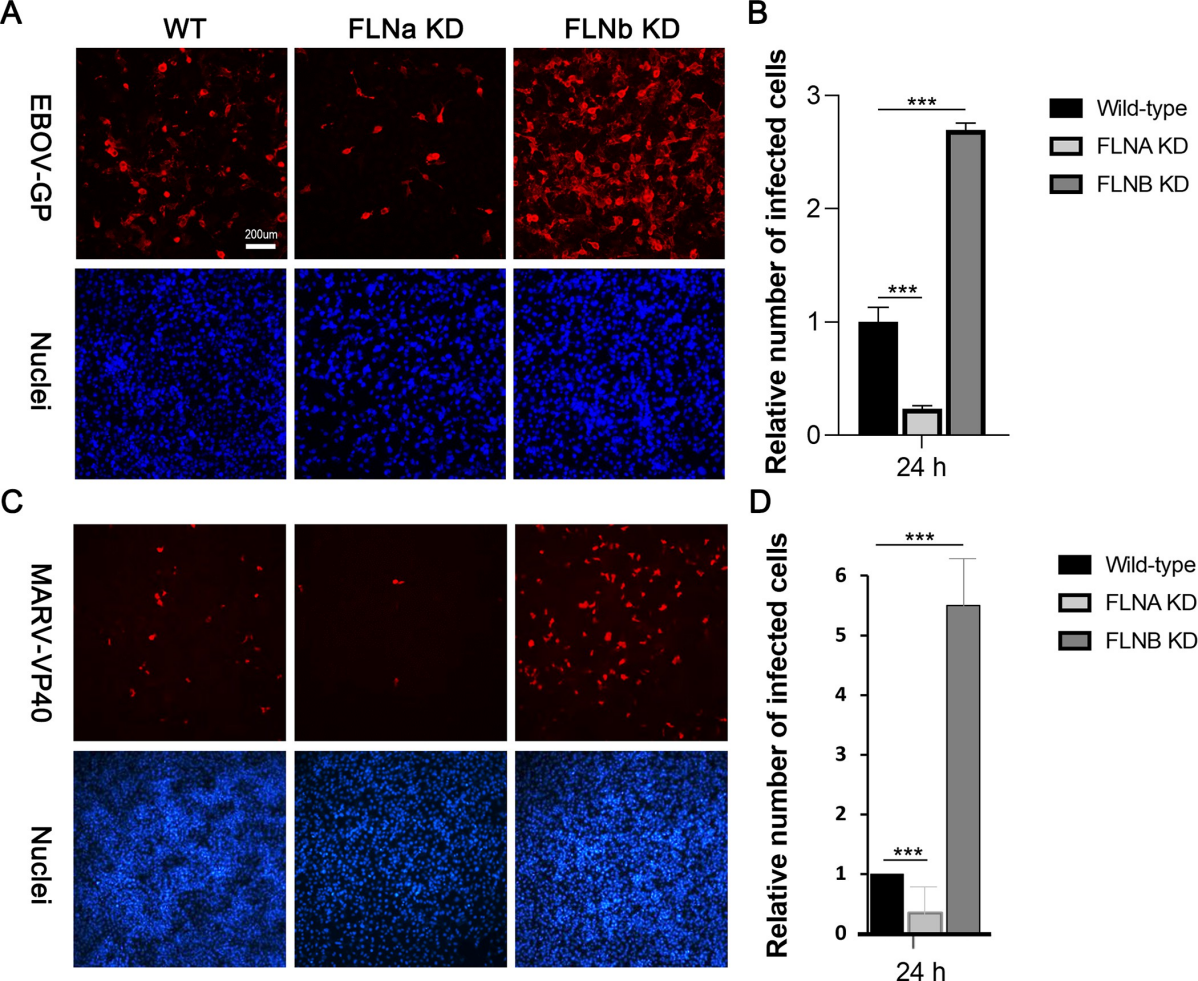
Filamin proteins regulate filovirus infectivity. (A) Representative images of WT HT-1080, FLNaKD and FLNbKD cells infected with EBOV at MOI = 0.1 for 24 hours and treated with anti-eGP antibody to detect infected cells (red) and Hoechst dye to stain nuclei (blue). (B) Infection efficiency in WT HT-1080, FLNaKD and FLNbKD monolayers at 24 hours post-EBOV infection was determined as the number of infected cells/number of nuclei. The numbers of infected cells in FLNKD samples are reported relative to the number in WT cells and are averages ± standard deviations of 3 independent experiments. (C) Representative images of WT HT-1080, FLNaKD and FLNbKD cells infected with MARV at MOI = 0.1 for 24 hours and treated with anti-mVP40 antibody to detect infected cells (red) and Hoechst dye to stain nuclei (blue). (D) Infection efficiency in WT HT-1080, FLNaKD and FLNbKD monolayers at 24 hours post-MARV infection was determined as the number of infected cells/number of nuclei. The numbers of infected cells in FLNKD samples are reported relative to the number in WT cells and are averages ± standard deviations of 6 independent experiments for FLNaKD samples and 3 independent experiments for FLNbKD samples. A one-way ANOVA followed by Dunnett’s multiple comparison test for one variable was used to assess a statistical difference between infection efficiencies in WT HT-1080 and FLNaKD or FLNbKD cells. *** = p value <0.0001 was determined for each sample pair analyzed.

We then infected WT HT-1080, FLNaKD, and FLNbKD cells with authentic EBOV (multiplicity of infection [MOI] = 0.1) and stained for eGP expression in infected cells at 24 hours post infection (Fig 2A). We observed that EBOV infectivity in FLNaKD cells was decreased significantly, whereas EBOV infectivity in FLNbKD cells was enhanced significantly as compared to WT HT-1080 control cells (Fig 2A). We quantified the number of infected cells in multiple independent experiments as shown in Fig 2B.

We also infected WT HT-1080, FLNaKD, and FLNbKD cells with authentic MARV (MOI = 0.1) and stained for mVP40 expression in infected cells at 24 hours post infection (Fig 2C). We also observed that MARV infectivity in FLNaKD cells was decreased significantly, although to a lesser extent than EBOV. Additionally, we observed that MARV infectivity in FLNbKD cells was enhanced significantly as compared to WT HT-1080 control cells (Fig 2C). We again quantified the number of infected cells in multiple independent experiments as shown in Fig 2D. Somewhat surprisingly, these findings suggested that FLNa and FLNb had opposite effects on filovirus infectivity.

### Filamin proteins regulate entry of psVSV-RFP-eGP pseudotypes

**Fig 3:**
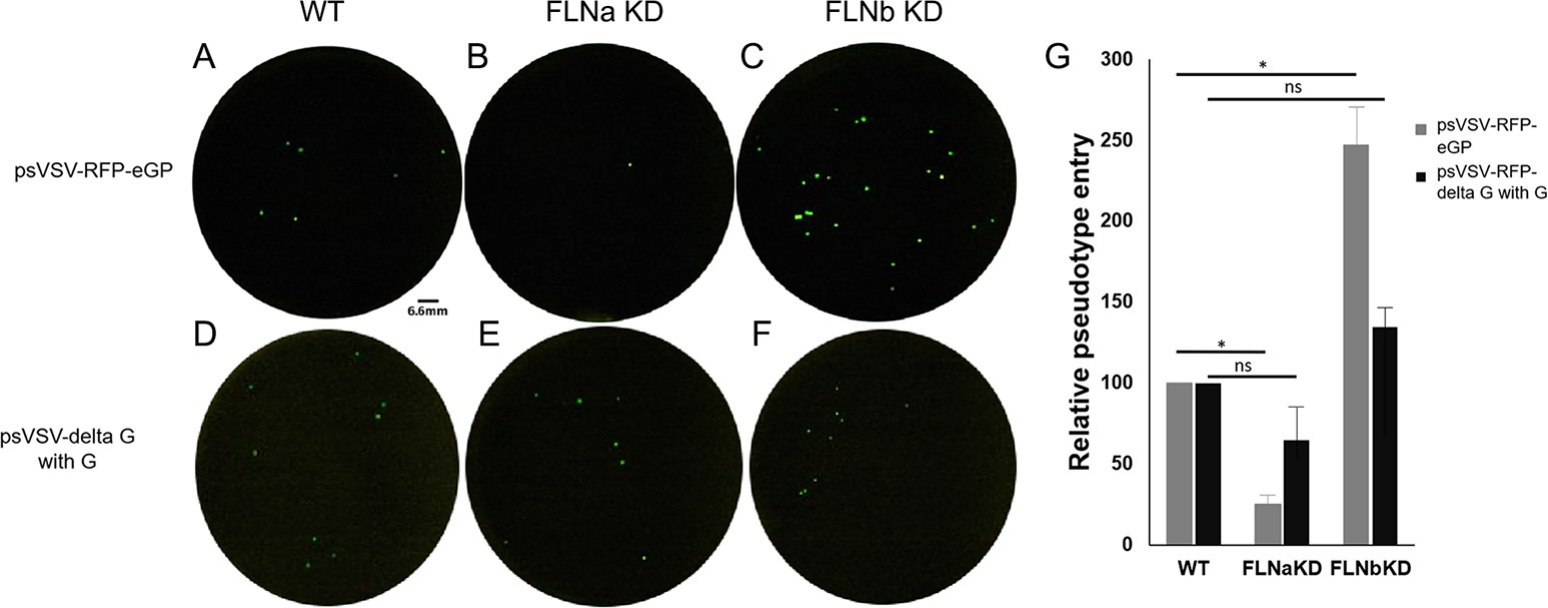
Filamin proteins regulate entry of psVSV-RFP-eGP pseudotypes. (A-C) Representative images of psVSV-RFP-eGP pseudotype particle entry (MOI = 3) into HT1080-WT, FLNaKD, and FLNbKD cells, with psVSV-RFP-eGP positive cells shown in green within a single representative 96 well. (D-F) Representative images of psVSV-RFP-deltaG with G particle entry (MOI = 1) into HT1080-WT, FLNaKD, and FLNbKD cells, with psVSV-RFP-deltaG with G positive cells shown in green within a single representative 96 well. (G) Graph showing the average percent of psVSV-RFP-eGP particle entry, as compared to psVSV-RFP-deltaG with G particle entry, relative to HT1080-WT cells at 24 hour post transduction from 3 independent experiments. Statistical analysis was performed using a 2-sample student t test; * = p value <0.05, ns = not significant.

To determine whether the distinct roles of FLNa and FLNb knockdown on live filovirus infectivity could be specificially linked to virus entry mediated by the surface glycoprotein, we used VSV pseudotypes expressing EBOV eGP (psVSV-RFP-eGP). WT HT-1080, FLNaKD or FLNbKD cells were transduced with psVSV-RFP-eGP pseudotypes expressing red flourescent protein (RFP), and RFP^+^ cells were visualized and quantified at 24 hrs post-transduction. In repeated experiments, we observed that entry of psVSV-RFP-eGP pseudotypes, as determined by quantification of RFP^+^ cells, was reduced significantly in FLNaKD cells as compared to that observed in WT HT-1080 cells (Fig 3). In agreement with live virus data, entry of psVSV-RFP-eGP particles was enhanced significantly in FLNbKD cells as compared to that in WT HT-1080 control cells (Fig 3). We did not observe any significant inhibition or enhancement of entry of VSV G-containing pseudtoypes (psVSV-deltaG with G) into FLNaKD or FLNbKD cells (Fig 3G), likely due to mechanistic differences in entry of VSV G-containing particles (via endocytosis; [29,30] vs. EBOV GP-containing particles (via macropinocytosis; [7]. In sum, the phenotypes observed for entry of psVSV-RFP-eGP pseudotypes into FLNa and FLNb knockdown cells closely mimicked those observed for infectivity of live filoviruses, suggesting that expression of endogneous FLNa and FLNb positively and negatively regulate filovirus GP-mediated entry, respectively.

### Filamin proteins regulate infectivity of infectious VSV recombinant (rVSV-eGP-mCherry) virus

**Fig 4:**
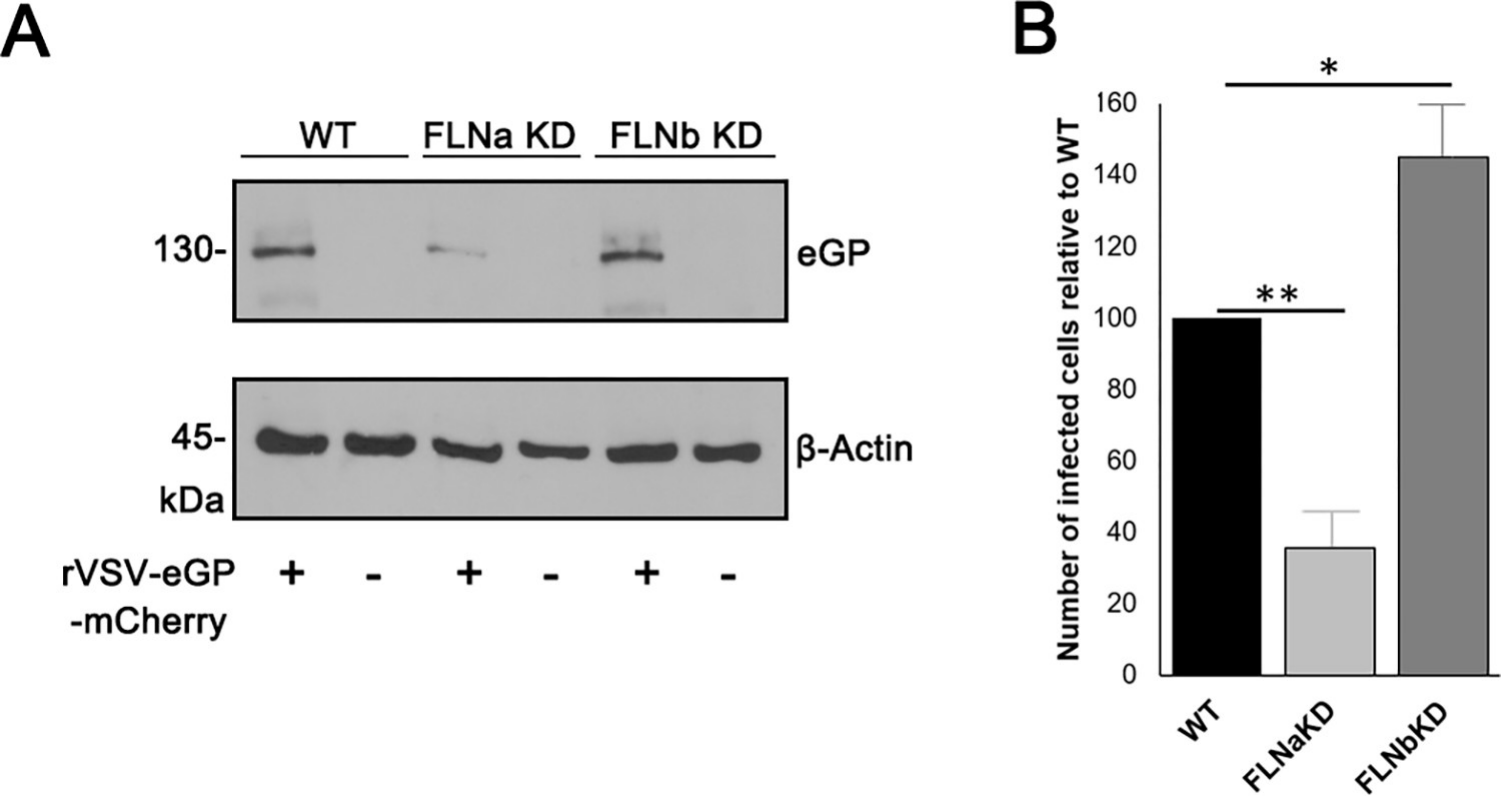
Filamin proteins regulate infectivity of infectious recombinant rVSV-eGP-mCherry. (A) Representative Western blot showing the expression of the indicated proteins in HT-1080 WT, FLNaKD, or FLNbKD cells mock infected or infected with recombinant virus rVSV-eGP-mCherry. (B) Bar graph showing the average relative entry of recombinant rVSV-mCherry-eGP from 5 independent experiments. Statistical analysis was performed using 2 sample-student t test, * = p value <0.05, ** = p value <0.01.

In a complementary approach, we asked whether infectivity of an infectious VSV recombinant virus was affected in the FLN knockdown cells in a manner similar to that of live EBOV and MARV and the psVSV-RFP-eGP pseudotypes. We utilized a replication competent VSV recombinant virus (rVSV-eGP-mCherry) that encodes EBOV eGP on its surface in place of VSV G, and also encodes mCherry in a separate ORF as a marker of infectivity. WT HT-1080, FLNaKD, and FLNbKD cells were either mock-infected, or infected with rVSV-mCherry-eGP for 10 hours, and viral infectivity was quantified by fluorescence microscopy (Fig 4). To confirm infection, we detected eGP protein by Western blotting specifically in virus infected cells, and the levels of eGP were reduced in FLNaKD cells as compared to those in control and FLNbKD cells (Fig 4A). Quantification of mCherry positive cells from five independent experiments revealed a similar trend as described above, in that infectivity of rVSV-eGP-mCherry was decreased in FLNaKD cells, whereas infectivity was enhanced in FLNbKD cells as compared to infectivity in WT HT-1080 cells (Fig 4B). These data are consistent with those described above using authentic EBOV and MARV and the psVSV-RFP-eGP pseudotypes, and thus we conclude that FLNa and FLNb proteins distinctly regulate GP-mediated infectivity and entry into HT-1080 cells.

### Filamin proteins regulate entry of psVSV-RFP-eGP pseudotypes into Hela cells

**Fig 5:**
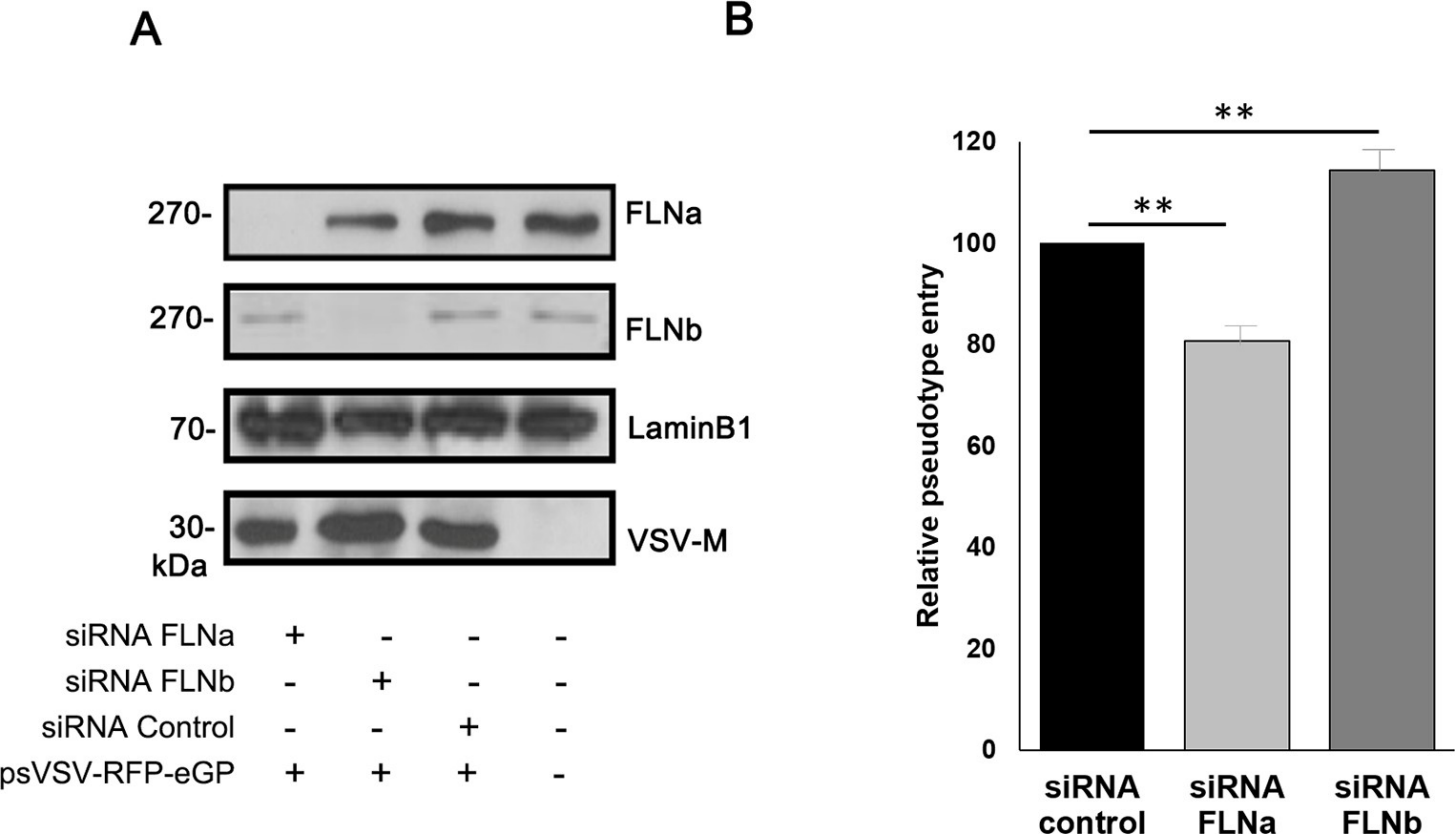
siRNA assay to assess entry of psVSV-RFP-eGP pseudotype into HeLa cells. (A) Representative Western blot showing detection of the indicated proteins in cells receiving no siRNA, control siRNA, FLNa-specific siRNA, or FLNb-specific siRNA. Cells were non-transduced, or transduced with psVSV-RFP-eGP pseudotypes as shown. (B) Bar graph showing relative psVSV-RFP-eGP particle entry into FLNKD cells as compared to WT cells in 4 independent experiments. Statistical analysis was performed using 2-sample student t test; ** = p value < .01.

To demonstrate that the effect of FLN proteins on filoviral infectivity/entry was not unique to the HT-1080 cell line, we used siRNA to knockdown expression of endogenous FLNa and FLNb in Hela cells, followed by transduction with psVSV-RFP-eGP pseudotypes to assess entry. Briefly, HeLa cells received non-specific siRNA, FLNa-specific siRNA, or FLNb-specific siRNA followed by transduction with psVSV-RFP-eGP pseudotypes (Fig 5A). We found that entry of psVSV-RFP-eGP particles was inhibited in FLNa siRNA knockdown cells, whereas entry of psVSV-RFP-eGP particles was enhanced in FLNb siRNA knockdown cells as compared to non-specific siRNA controls (Fig 5B). These results suggest that the regulatory effects of FLNa and FLNb proteins observed for live filoviral infectivity/entry is not unique to the HT-1080 cells, and further highlights the disparate mechanisms by which FLNa and FLNb regulate filoviral GP-mediated entry.

### Filamin proteins regulate infectivity of EBOV and MARV in primary human macrophages

**Fig 6:**
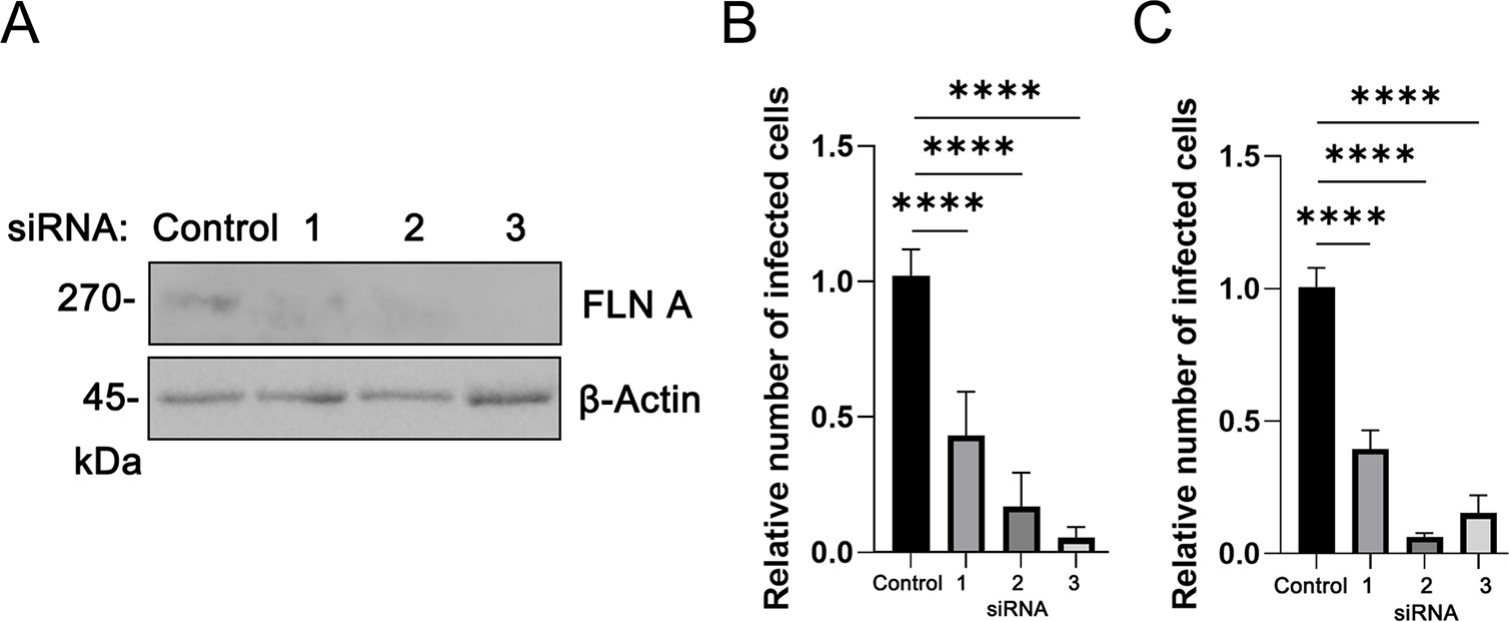
siRNA knockdown to assess infectivity of authentic EBOV and MARV in primary human macrophages. (A) Representative Western blot showing detection of the indicated proteins in macrophages receiving control siRNA or three individual FLNa-specific siRNAs (1, 2, and 3). (B) Bar graph showing the relative number of infected cells to non-specific siRNA controls after infection with EBOV-GFP (MOI = 0.2) at 24 hours post infection in 3 independent experiments. (C) Bar graph showing relative number of infected cells to non-specific siRNA controls after infection with MARV (MOI = 0.2) at 24 hours post infection in 3 independent experiments. A one-way ANOVA followed by Dunnett’s multiple comparison test for one variable was used to assess a statistical difference between infection efficiencies in cells transfected with non-specific siRNA vs. FLNa-specific siRNAs. **** = p value <0.0001 was determined for each sample pair analyzed.

Macrophages are known to be a primary and early target cell for EBOV and MARV infection [31,32]. Therefore, we sought to determine whether knockdown of FLNa would affect filovirus infection of primary human macrophages. We transfected FLNa-specific or non-specific siRNA into monocyte-derived macrophages (MDMs) and then infected with authentic EBOV or MARV. We did acheive efficient knockdown of endogenous FLNa with three individual FLNa-specific siRNAs (Fig 6A), as compared to a non-specific siRNA control. Notably, both EBOV and MARV infectivity was significantly inhibited in primary human macrophages treated with FLNa-specific siRNAs compared to that in control siRNA treated cells (Fig 6B and 6C). These results further validate the role of endogenous FLNa in regulating entry of EBOV and MARV into a biologically relevant primary human target cell.

### Filamin proteins regulate macropinocytosis

**Fig 7:**
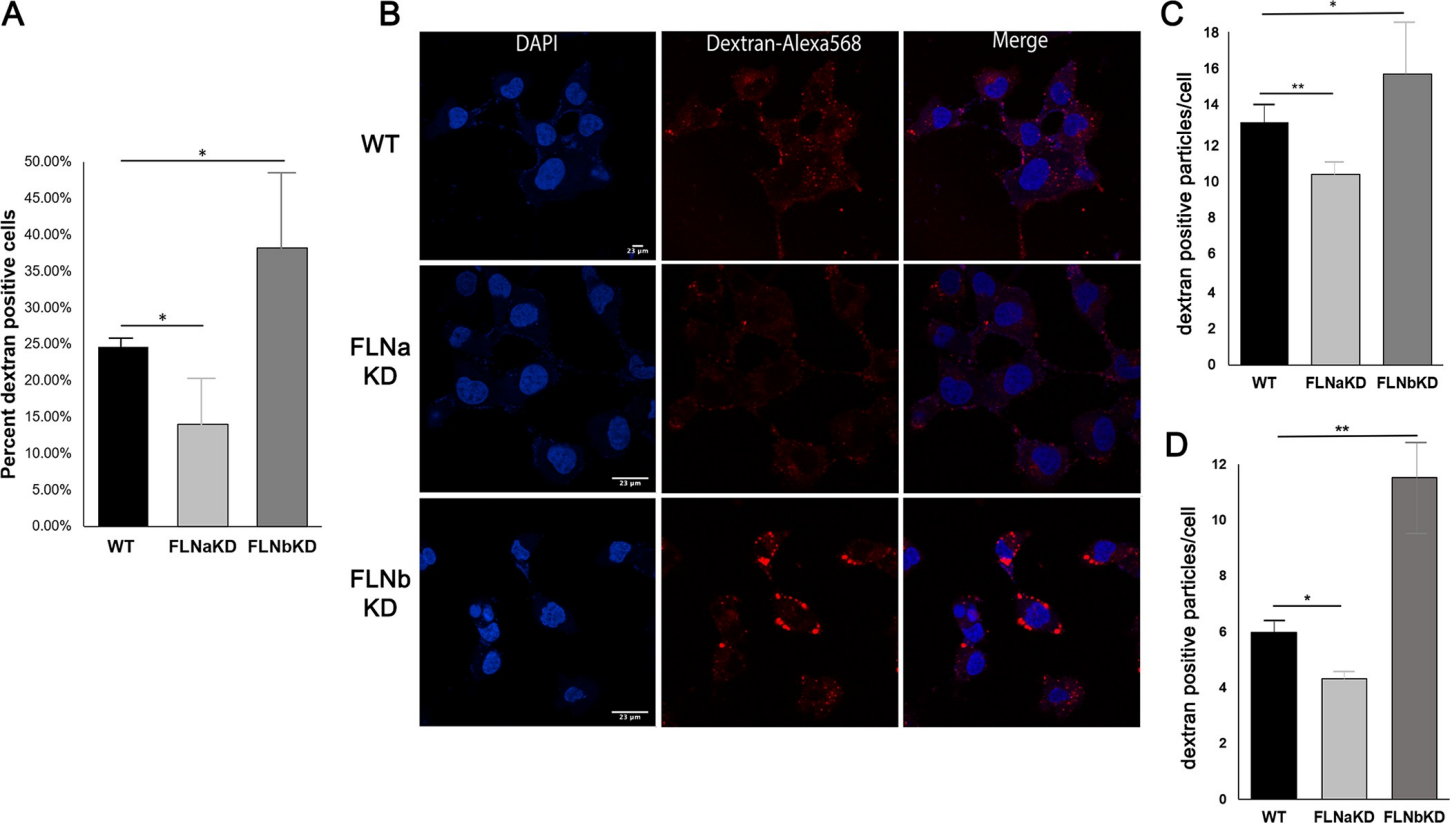
**Differing rates of uptake by macropinocytosis observed in FLN KD cells as compared to HT1080-WT cells** (A) Graph showing average percent dextran-Alexa568 (MW 10K) positive cells after 5 minutes of co-incubation with EGF in 3 independent experiments by flow cytometry. Statistical analysis performed using 2-sample student t test; * = p value <0.05 (B) Representative confocal images showing nuclei stained with Hoechst (blue) and co-incubated with dextran-Alexa568 (MW 10K) and EGF (red) of HT1080-WT (top row), FLNaKD (middle row) and FLNbKD cells (bottom row). (C) Graph showing average number of dextran-Alexa568 (MW 10K) positive particles per cell in HT1080-WT and FLNKD cells fixed after 5 minutes of co-incubation with EGF (from 5 independent experiments). Statistical analysis was performed using 2-sample student t test; * = p value <0.05, ** = p value <0.01 (D) Graph showing average number of dextran-TRITC (MW 70K) positive particles per cell in HT1080-WT and FLNKD cells fixed after 30 minutes of co-incubation with EGF (from 3 independent experiments). Statistical analysis was performed using 2-sample student t test; * = p value <0.05, ** = p value <0.01.

Filovirus entry is mediated primarily by macropinocytosis [7], an actin-dependent endocytosis mechanism that allows cellular uptake of extracellular fluids and soluble macromolecules into large vacuoles. Thus, we reasoned that the mechanism by which the FLN proteins regulate infectivity/entry of EBOV and MARV may involve their ability to modulate the process of macropinocytosis. To determine whether the observed differences in filovirus entry in the FLNKD cells may be due, in part, to the effect of filamin-regulated macropinocytosis, we used flow cytometry to measure fluid-phase macropinocytosis of fluorescently-tagged 10K dextran-Alexa568 into endothelial growth factor (EGF)-stimulated FLNKD and WT cells at 5 minutes post incubation (Fig 7A). We used the 10K dextran-Alexa568 and a 5 minute incubation period to decrease the likehood of quantifying false positives of endosomes internalized by a clathrin-mediated pathway, as has been reported previously [33–36] Notably, we observed that the percent of fluorescent dextran taken up via macropinocytosis was reduced significantly in the FLNaKD cells as compared to that in WT HT-1080 cells (Fig 7A). In contrast, we observed that the percent of fluorescent dextran taken up via macropinocytosis was significantly enhanced in the FLNbKD cells compared to that taken up by WT HT-1080 cells (Fig 7A).

We used confocal microscopy to quantify internalized macropinosomes containing 10K dextran-Alexa568 in FLNKD vs HT-1080 WT cells at 5 minutes post incubation. Representative confocal microscopy images of WT HT-1080, FLNaKD and FLNbKD cells incubated with 10K dextran-Alexa568 and EGF are shown (Fig 7B). The bar graph represents the average number of dextran positive punta/cell from 5 independent experiments (Fig 7C). Our results show that the FLNaKD cells contained significantly fewer dextran positive macropinosomes than did the WT HT-1080 cells, whereas the FLNbKD cells contained significantly more dextran positive macropinosomes than did the WT HT-1080 cells.

In a complementary approach to further ensure that we were measuring uptake of dextran particles by macropinocytosis, we used confocal microscopy to quantify macropinosomes containing 70K dextran-Tetramethylrhodamine (TRITC) into FLNKD and HT-1080 WT cells. The 70K dextran-TRITC has been reported to enter cells via clathrin- and dynamin-independent macropinocytosis [37,38]. Similar to our previous findings, the FLNaKD cells contained significantly fewer 70K dextran positive macropinosomes than did the WT HT-1080 cells, whereas the FLNbKD cells contained significantly more 70K dextran positive macropinosomes than did the WT HT-1080 cells (Fig 7D). Together, these data suggest that the mechanism by which the FLNa and FLNb proteins inversely regulate filovirus entry may reflect their ability to similarly regulate the process of macropinocytosis at the plasma membrane.

## Discussion

Filoviruses rely on the integrity and dynamics of the host cytoskeletal architecture at the plasma membrane to facilitate entry into host cells. EBOV and MARV GPs are the viral proteins responsible for mediating attachment, entry, and fusion into target cells. However, a growing list of host cell proteins recently identified may also be important facilitators of these early events of the filovirus lifecycle. For example, DC-SIGN/L-SIGN, LSECtin, hMGL, β1-integrin and Tyro3 family receptors have been implicated as attachment factors, and cellular receptors like TIM-1 may function as a signal to trigger the macropinocytosis program required to internalize EBOV virions following GP attachment [7,13,14,21,39–41]. Macropinocytosis is characterized by actin-dependent membrane ruffling associated with the formation of macropinosomes of approximately 0.5–10 μm in diameter. Macropinocytosis and the dynamic flexibility of the actin cytoskeleton beneath the plasma membrane enable the cell to take up large substances, such as the long, filamentous filovirus virions [21]. Here we have identified actin-crosslinking filamin proteins A and B as novel regulators of filovirus entry/infectivity. Intriguingly, FLNa and FLNb appear to have opposing effects on EBOV and MARV entry, such that expression of FLNa is important for efficient filovirus entry whereas expression of FLNb may act as a barrier to restrict or negatively regulate entry. When we investigated the roles of FLNa and FLNb in regulating macropinosome formation in HT-1080 cells, we observed the same opposing effects on macropinocytosis as we did for EBOV and MARV entry. Notably, knockdown of endogenous FLNa reduced the efficiency of cellular uptake of two dextran markers, whereas knockdown of endogenous FLNb enhanced uptake of the same markers of macropinocytosis. Mechanistically, these findings suggest that FLNa and FLNb likely modulate filovirus entry by regulating the process of macropinocyctosis.

The opposite effects of FLNa and FLNb on entry and infectivity of EBOV and MARV was somewhat surprising since the filamin proteins share a high degree of homology between the conserved exon/intron structure. These two filamin isoforms physically interact and heterodimerize, suggesting a mechanism to regulate each other’s function, with loss of expression of one leading to upregulation of the other [19,42]. However, an antagonistic relationship between FLNa and FLNb occurs during the process of cell migration and spreading, whereby they differentially regulate the RhoA GTPase. RhoA is directly involved in promoting cell protrusions and macropinocytosis, with increased activity at the cell plasma during vesicle closure [19,43]. Additionally, we know from the literature that FLNa co-localizes with host protein Phafin2 on forming macropinosomes and dissociates together with actin. Phafin2 is expressed abundantly in dendritic cells and helps to strip away the dense actin/filamin coat from internalized macropinosomes allowing them to efficiently traffic through the cell to form the mature endosomal form [34]. Thus, the potential role and opposing effects of FLNa and FLNb on filovirus entry may indeed be linked to the process of macropinocytosis.

Alternatively, there are other filamin interactors that could be linked to the filovirus lifecycle and affect entry and infectivity of the virus. For example, FLNa promotes entry of HIV-1 by linking viral receptors to the actin cytoskeleton and by modulating the antiviral factor tetherin [44,45]. In addition, FLNa interacts with viral NS3 and NS5A proteins in chronic hepatitis C virus (HCV) infection, and can act as an adapter protein to the adenoviral and coxsackieviral receptor, Integrin β1 [44,46,47]. It is tempting to speculate that a FLNa-β1 integrin interaction may play a role in filovirus entry since β1 integrin expression has been linked to eGP-mediated entry of EBOV [41,48,49]. Alternatively, RNAse-L is a well-known member of the host innate immune system that interacts with FLNa to form a barrier to entry of Sendai virus [50,51]. Based on our findings, one could speculate that a similar restrictive barrier to filovirus entry may result from an RNAse-L/FLNb interaction. Furthermore, it is known that FLNb, but not FLNa, plays a scaffolding role in IFN signaling and can be modified by IFN-stimulated gene 15 (ISG15) [23]. Whether or not the effect of FLNb knockdown on enhancing EBOV and MARV infectivity may be due, in part, to FLNb-mediated regulation of antiviral immune defenses remains to be determined [22,23,52].

Our identification of FLNa and FLNb proteins as novel regulators of plasma membrane driven stage of EBOV and MARV entry provide new insights into the complex roles that host proteins play in regulating the filovirus lifecycle. A better understanding of this interplay between filoviral proteins and FLN proteins will be critical for our overall understanding of the biology and pathogenesis of filoviruses and other emerging pathogens, as well as for the future development of effective, host-directed antiviral therapies. Further investigation into possible pharmalogical inhibition of FLNa activity during early filovirus infection, for example, may benefit the development of a broad-spectrum, multifunctional antiviral therapeutic.

## Material and methods

### Cell lines, plasmids, and reagents

HeLa, HEK293T, HT-1080 WT, FLNaKD and FLNbKD cells were maintained in Dulbecco’s modified Eagle’s medium (DMEM) (Corning) supplemented with 10% fetal bovine serum (FBS) (Gibco), penicillin (100 U/mL)/streptomycin (100 μg/mL) (Invitrogen). Cells were grown at 37°C in a humidified 5% CO_2_ incubator. FLNaKD and FLNbKD cells were generated from parental HT-1080 cells as described previously [15]. The primary antibodies used in this study include mouse anti-FLNa (Santa Cruz), rabbit anti-FLNb antibody (Millipore), rabbit anti-LaminB1 (Abcam), rabbit anti-eGP (Invitrogen), mouse anti-GFP (Rosche) and mouse anti-β-actin (Proteintech). Dextran-Alexa568 (MW 10K) and Dextran-TRITC (MW 70K) were purchased from Invitrogen. VSV M protein was detected using mouse anti-VSV-M monoclonal antibody 23H12 (kindly provided by D. Lyles, Wake Forest, Winston-Salem, NC, USA). FLNa-specific siRNA, FLNb-specific siRNA and control siRNA pools were purchased from Santa Cruz and Origene. The rVSV-eGP-mCherry virus [53] was kindly provided by P. Bates (UPenn School of Medicine).

### MTT assay

Cell proliferation was determined by using the 3-(4,5-dimethylthiazol-2-yl)-2,5-diphenyltetrazolium bromide (MTT) colorimetric assay. Briefly, cells were seeded in 96-well plates and incubated for 24, 48, and 72 hours, and cells were washed with phosphate-buffered saline (PBS) and then incubated in MTT solution for 3 hours. After dimethyl sulfoxide was added into each well, the absorbance was measured at 490 nm to determine cell viability with a microplate reader.

### EBOV and MARV infection and staining

Experiments with live viruses were performed in the BSL-4 laboratory at Texas Biomedical Research Institute (Texas Biomed, San Antonio, TX) in accordance with standard operating procedures and protocols approved by the Institute’s Biohazard & Safety and Recombinant DNA Committees. The NCBI accession numbers for filoviruses used in these studies were NC_002549 (EBOV variant Mayinga), KF990213 (recombinant EBOV variant Mayinga, encoding GFP), and NC_001608 (Marburg virus strain Musoke). The virus stocks were obtained from the Texas Biomed repository, grown as before [54,55], and viral titers were determined using standard plaque assays.

Briefly, HT-1080 WT and FLNKD cells grown in 96-well plates were incubated with EBOV or MARV at MOI = 0.1 in triplicate for 1 hour, then washed and overlaid with fresh medium. After 24 hours, cells were fixed and were treated with an antibody to viral GP (clone 4F3; IBT Bioservices) or mVP40 (IBT Bioservices) to detect infection and Hoechst 33342 dye (Thermofisher) to stain nuclei. Sample photographs acquired by Nikon Ti-Eclipse microscope (Nikon, Tokyo, Japan) were analyzed by CellProfiler software (Broad Institute) to determine infection efficiency, calculated as the number of infected (GP-positive or mVP40-positive) cells over the total number of cells (nuclei), for each condition.

Each experiment was repeated three times. A one-way ANOVA followed by Dunnett’s multiple comparison test for one variable was used to assess a statistical difference between infection efficiencies in WT and FLNKD cells. A p value was determined for each sample pair analyzed, and the difference was considered statistically significant if p<0.05.

### psVSV-RFP-eGP propagation

psVSV-RFP-eGP pseudotyped particles were generated using a VSV platform that incorporates the EBOV GP protein into VSV envelopes by transfection of HEK293T cells with pCG1 EBOV GP expression plasmid (kindly provided by P. Bates, UPenn School of Medicine). At 30 hours post transfection, the eGP expressing cells were transduced for 4 hours with psVSV-RFP-deltaG pseudotyped with G (kindly provided by P.Bates, UPenn School of Medicine). At 28–30 hours post transduction, the media containing the psVSV-RFP-eGP pseudotype particles was harvested and clarified by centrifugation twice at 4,000 rpm for 15 minutes. Viral particles were additionally clarified by ultracentrifugation at 36,000rpm for 2 hours and then stored at −80°C until use. Both psVSV-RFP-deltaG pseudotyped with G and psVSV-RFP-eGP express RFP.

### rVSV-eGP-mCherry infection

HT-1080 WT and FLNKD cells were seeded at 1 ×10^6^ cells overnight in 6-well plates. rVSV-eGP-mCherry (MOI = 0.1 ~5 ×10^7^ RFU /ml) was added to cells for 1 hour at 37°C, virus inoculum was removed, and the cells were washed once with 1× DPBS. DMEM with 1% methylcellulose was added to the cells at 37°C for 10 hours. Cells were washed 3 times with DPBS and either harvested for Western blotting or fixed with 4%PFA for 15mins at room temperature. Unfixed cell extracts were harvested with RIPA and the indicated proteins were detected by Western blotting. Briefly, unfixed cells were harvested and lysed RIPA buffer and clarified for 5 min at 15,000 rpm. Cell lysates were suspended in loading buffer with boiling, fractionated by SDS-PAGE and EBOV GP and actin proteins were detected using specific antisera. For fixed cells, mCherry expression was visualized and quantified on a fluorescent microscope.

### siRNA knockdown and psVSV-RFP-eGP transduction

HeLa cells in Opti-MEM (Thermofisher) in 6-well plates were transfected twice with either control siRNAs or FLNa-specific or FLNb-specific siRNAs (siRNA pools purchased from Santa Cruz Biotechnology) at a final concentration of 50 nM by using Lipofectamine Invitrogen at 2-day intervals. A total of 1.0 μg of eVP40 DNA was transfected with the second round of siRNAs. HeLa cells were then transduced with psVSV-RFP-eGP pseudotypes at MOI = 1 (~1×10^6^ red fluorescent units per well) added to Opti-MEM and incubated with cells for 1 hour at 37°C. Pseudovirus inoculum was removed, and the cells were washed once with 1× DPBS. RFP expression was visualized and quantified on a fluorescent microscope at 24 hours post transduction. Cell extracts were then harvested and the indicated proteins were detected in cell samples by Western blotting.

### Primary human macrophages

Peripheral blood was collected from healthy adult human donors according to the University of Texas Health-approved IRB protocol 20180013HU to prepare monocyte-derived macrophages (MDMs) as we described previously [56]. MDMs were cultured in RPMI medium supplemented with 10% autologous serum at 37°C in a humidified 5% CO2 incubator.

### siRNA treatments in MDMs and immunoblotting

To deplete MDMs of FLNa, cells plated in 96-well or 12-well plates were transfected with three individual siRNA duplexes targeting the gene to a final concentration of 25 nM of FLNa siRNAs or the same concentration of a non-specific siRNA control (Origene). The transfections were performed in triplicate, using TransIT-X2 reagent (Mirus), following the manufacturer’s recommendations. After 48 hours, the siRNA treatments in 96-well plates were removed, and the MDMs were infected with EBOV-GFP or MARV at a MOI of 0.2 for 24 hours. The cells were stained and analyzed as above. FLNa protein depletion was assessed by immunoblotting as previously described [55].

### Macropinocytosis assay

HT-1080 WT and FLNKD cells were seeded overnight in 12-well plates and then media was replaced with Opti-MEM without phenol-red for 3 hours at 37°C. 5ul of DMSO was added to negative control 30 mins prior to addition of 0.5 mg/ml of 10 K dextran-Alexa568 (Invitrogen) with 100 ng/ml of endothelial growth factor (EGF) for 5 minutes at room temperature. Cells were washed twice with cold 1× DPBS and fixed with IC Fixation buffer (Invitrogen) for 10 minutes at room temperature. Cells were collected in DMEM with 1% FBS at 4°C overnight. Flow cytometry was performed with LSRFortessa (BD Biosciences) and data analyzed using FlowJo software (FlowJo, LLC). For confocal microscopy, cells were seeded on 35mm MatTek dishes and treated as previously described with 0.1mg/ml of dextran-Alexa568 (MW 10K) and 100ng/ml of EGF. Cells were fixed with 4% PFA and stained with Hoechst. Images were acquired using a laser scanning confocal microscope (Leica SP5-FLIM Inverted), equipped with 63× oil immersion objective. Images were processed using Fiji and 3–5 fields per sample were randomly selected with 5–10 cells per visual field for dextran particle analysis. HT1080-WT and FLNKD cells were fixed in 4 well chamber cover glass slides (Cellvis) after 30 minutes of co-incubation with 1.0 mg/ml dextran-TRITC (MW 70K) and 20 ng/ml EGF. Images were acquired using a laser scanning confocal microscope with 100× oil immersion objective and 6–7 fields per sample were randomly selected with 5–25 cells per visual field for dextran particle analysis by Fiji. In brief, red channel was converted to 8-bit grey scale and background subtraction was used after threshold fluorescence was set. Size filter was set to an area of 0.2–20.0 μm^2^ to exclude non-macropinosome structures for particle counts [33], and Hoechst staining of nuclei was used to calculate average number of dextran^+^ particles per cell within an image.
